# Genetics of Exertional Heat Illness: Revealing New Associations and Expanding Heterogeneity

**DOI:** 10.3390/ijms252011269

**Published:** 2024-10-19

**Authors:** Nyamkhishig Sambuughin, Ognoon Mungunsukh, Michael G. Klein, Mingqiang Ren, Peter Bedocs, Josh B. Kazman, Kristen Cofer, Liam P. Friel, Beth McNally, Kyung Kwon, Mark C. Haigney, Jeffrey C. Leggit, Marzena Pazgier, Patricia A. Deuster, Francis G. O’Connor

**Affiliations:** 1 Consortium for Health and Military Performance, Department of Military and Emergency Medicine, F. Edward Hébert School of Medicine, Uniformed Services University, Bethesda, MD 20814, USA; mingqiang.ren.ctr@usuhs.edu (M.R.); josh.kazman.ctr@usuhs.edu (J.B.K.); kristen.cofer.ctr@usuhs.edu (K.C.); liam.friel.ctr@usuhs.edu (L.P.F.); beth.mcnally.ctr@usuhs.edu (B.M.); kyung.kwon.ctr@usuhs.edu (K.K.); jeff.leggit@usuhs.edu (J.C.L.); patricia.deuster@usuhs.edu (P.A.D.); francis.oconnor@usuhs.edu (F.G.O.); 2 Henry M. Jackson Foundation for the Advancement of Military Medicine, Inc., Bethesda, MD 20817, USA; mungunsukh.ognoon.ctr@usuhs.edu (O.M.); peter.bedocs.ctr@usuhs.edu (P.B.); 3 Department of Anatomy Physiology and Genetics, F. Edward Hébert School of Medicine, Uniformed Services University, Bethesda, MD 20814, USA; 4 Military Cardiovascular Outcomes Research, Cardiology Division, Department of Medicine, F. Edward Hébert School of Medicine, Uniformed Services University, Bethesda, MD 20814, USA; michael.klein@usuhs.edu (M.G.K.); mark.haigney@usuhs.edu (M.C.H.); 5 Defense & Veterans Center for Integrative Pain Management, Department of Anesthesiology, F. Edward Hébert School of Medicine, Uniformed Services University, Bethesda, MD 20814, USA; 6 Department of Family Medicine, F. Edward Hébert School of Medicine, Uniformed Services University, Bethesda, MD 20184, USA; 7 Infectious Disease Division, Department of Medicine, F. Edward Hébert School of Medicine, Uniformed Services University, Bethesda, MD 20184, USA; marzena.pazgier@usuhs.edu

**Keywords:** exertional heat illness, whole-exome sequencing, rare variant, variant pathogenicity, genetic heterogeneity

## Abstract

Environmental heat stress represents a pervasive threat to warfighters, athletes, and occupational workers, impacting performance and increasing the risk of injury. Exertional heat illness (EHI) is a spectrum of clinical disorders of increasing severity. While frequently predictable, EHI can occur unexpectedly and may be followed by long-term comorbidities, including cardiovascular dysfunction and exercise intolerance. The objective of this study was to assess genetic factors contributing to EHI. Whole-exome sequencing was performed in a cohort of 53 cases diagnosed with EHI. Rare variants in prioritized gene sets were analyzed and classified per published guidelines. Clinically significant pathogenic and potentially pathogenic variants were identified in 30.2% of the study cohort. Variants were found in 14 genes, including the previously known *RYR1* and *ACADVL* genes and 12 other genes (*CAPN3*, *MYH7*, *PFKM*, *RYR2*, *TRPM4*, and genes for mitochondrial disorders) reported here for the first time in EHI. Supporting structural and functional studies of the *TRPM4* p.Arg905Trp variant show that it impairs the thermal sensitivity of the TRPM4 channel, revealing a potentially new molecular mechanism contributing to EHI susceptibility. Our study demonstrates associations between EHI and genes implicated in muscle disorders, cardiomyopathies, thermoregulation, and oxidative phosphorylation deficiencies. These results expand the genetic heterogeneity of EHI and shed light on its molecular pathogenesis.

## 1. Introduction

Exertional heat illness (EHI) represents a spectrum of clinical conditions that may occur in response to strenuous exercise, most often in hot, humid environments. These disorders can progress in severity to include heat exhaustion; heat injury, which is defined as heat exhaustion with evidence of end organ injury; and exertional heat stroke (EHS) [1,2]. Recreational and competitive athletes, occupational laborers, and tactical athletes (military personnel, firefighters, and police) are particularly vulnerable, secondary to their level of exertion and environmental exposure. EHI is generally characterized by weakness and fatigue, core body hyperthermia, core temperature elevations above 40 °C in cases of EHS, and by signs of central nervous system (CNS) dysfunction [1,2,3]. If not recognized and treated promptly, EHS can potentially result in multi-organ failure and, in some cases, death. In fact, EHS is one of the leading causes of death in young, otherwise healthy competitive athletes and military personnel who engage in vigorous exercise [3]. The last several decades have witnessed an increase in heat wave frequency; accordingly, there is considerable speculation that anthropogenic climate change will continue to be a significant global health threat [4,5]. Climate change and associated heatwaves can increase mortality globally. Heat stress will affect cognitive function, as well as significantly reduce physical work and activity, especially in elderly populations, occupational workers, and tactical athletes with long-term health co-morbidities, including cardiovascular dysfunction and weakened immune system [1,4].

The etiology of EHI is multifactorial, with contributions from various environmental and genetic risk factors. Whereas environmental risk factors are well investigated, limited research has sought to characterize the inherent risks of susceptibility to EHI [1,3]. Studies have shown that variants in the ryanodine receptor 1 gene (*RYR1*) are associated with EHI [6,7,8,9]. *RYR1* encodes the skeletal muscle calcium-release channel, ryanodine receptor 1 (RyR1) [9,10]. This channel plays a central role in muscle Ca^2+^ regulation and links surface membrane potential to muscle contraction, a process known as excitation contraction coupling [10]. *RYR1* is a major gene for malignant hyperthermia (MH), a primarily autosomal dominant trait that manifests as a hypermetabolic reaction with hyperthermia, muscle rigidity, and tachycardia triggered by halogenated anesthetics [9]. Although not common, non-anesthesia-related environmental factors—such as exercise, heat, or both—may trigger MH [6,7,8,9]. The similarities between MH and EHI have led to the recent discovery of variants in new candidate genes, *TRPV1* and *ASPH*, in individuals with both conditions [11,12]. In addition, results from a cohort study of patients diagnosed with EHI proposed a possible role of variants in some genes encoding enzymes that maintain oxidative phosphorylation (OXPHOS) during exercise [8]. Despite these emerging genetic factors, the underlying causes of individual variability in susceptibility to EHI remain poorly defined.

Physical exertion challenges the cardiovascular system to simultaneously support increased blood flow to meet the metabolic demand of contracting muscle, and the cutaneous vasculature to dissipate body heat [1,13]. EHI develops when these competing events transition from a compensable state, whereby heat can dissipate, to a non-compensable state, whereby heat cannot dissipate adequately [13]. This physiological transition emphasizes the role of the cardiovascular response in EHI. Retrospective studies of young athletes and service members, including those who have suffered from severe EHI, have demonstrated impaired cardiac function or even sudden death due to underlying heart conditions [14,15]. Nevertheless, the inherent risks associated with cardiac muscle dysfunction have never been addressed in EHI, although the genetics of cardiomyopathies are well characterized [16,17,18].

The goal of this study was to characterize the clinical features of a series of EHI cases and identify potential genetic risk factors contributing to EHI susceptibility. Based on findings from previous genetic studies and the role of cardiac dysfunction in the development of EHI, we hypothesized that rare, deleterious coding variants in genes that are implicated in skeletal and cardiac muscle disorders of contraction, Ca^2+^ regulation and cellular energy metabolism would increase susceptibility to EHI. To test this hypothesis, we performed whole-exome sequencing (WES) in a cohort of 53 EHI cases.

## 2. Results

### 2.1. Study Cohort Characteristics

Demographic and clinical characteristics of the study cohort are summarized in Table 1. The study cohort was predominantly male, with a median age of 27 and a median peak core body temperature of 41.1 °C during EHI. The majority (56.6%) of the cohort experienced a single EHI event. However, 48.9% of the EHI cases had severe events with collapse or syncope and signs of CNS involvement. Rhabdomyolysis was present in 18 (38.3%) cases, with a mild increase in CK ranging from >2000–42,299 IU/L and associated muscle symptoms—muscle cramping, weakness, and pain. Nine EHI cases had marked increases in ALT and AST (>700 IU/L each), whereas six EHI cases had acute kidney injury, according to their medical records. Of six cases evaluated for MH, four tested positive for caffeine–halothane contracture tests (CHCT) and were diagnosed as MH susceptible. Other risk factors were sickle cell trait and family history of hypertension.

Environmental risk factors, including types of exertional activities and apparent temperature on the day of EHI events, are also presented in Table 1. Results show that running, followed by military training with full gear or heavy weight were frequent exercise activities performed by the study cases under a relatively hot environment.

### 2.2. WES Results and Variant Classification

To test our hypothesis, genes that encode proteins involved in skeletal muscle excitation contraction coupling and oxidative metabolism were prioritized and analyzed due to previous reports regarding their implication in EHI and exercise intolerance [1,6,7,8,9,10,11,12,19,20,21,22]. Analyses further focused on genes that encode proteins with similar functions in cardiac muscle, including sarcomere structure, contraction and calcium regulation, and those involved in the production of cellular energy by OXPHOS [8,15,16,17,18,20,21,22,23,24]. WES revealed 97 rare coding variants in 66 genes, which were grouped into categories based on associated diseases and disease mechanisms (Table 2 and Supplemental Tables S1–S3). All variants were in heterozygous states across the study cohort.

Twenty-eight NA variants were evaluated and classified per ACMG guidelines [25]. Among NA variants, three were loss-of-function (LoF) null variants with premature stop codons, and 25 were nonsynonymous variants. The null variants in *C1QBP* and *ETFB* were classified as LP, because LoF is a known mechanism of OXPHOS deficiencies that are caused by these genes [23]. The remaining NA variants, including another LoF variant in *MYH6* and other nonsynonymous variants, were classified as VUS based on associated disease mechanisms, variant frequencies, and metascores (Supplemental Tables S1–S3). As a result of these assessments, the number of variants with P/LP and VUS classifications increased to 8 and 89, respectively.

### 2.3. Pathogenic and Potentially Pathogenic Variants in EHI

Because of clinical significance, we prioritized variant pathogenicity and applied stringent criteria for metascores with high predictive values of variant pathogenicity for all identified nonsynonymous VUS. Five variants met the criteria for variant pathogenicity metascores and were considered potentially pathogenic. Despite their moderate metascores, we also considered variants in *CAPN3*, *TRPM4*, and *PPA2* as potentially pathogenic based on known classifications in ClinVar and therefore worthy of further investigation in this study. Overall, these analyses identified 16 (30.2%) EHI cases with disease-causing pathogenic and/or potentially pathogenic variants in 14 genes (Table 3). As shown, 3 cases carried variants in genes known to be implicated in EHI [6,7,8] and 13 cases carried variants in 12 genes that have not been reported previously in association with EHI.

Clinical features of EHI cases with these variants are given in Supplemental Table S4. Of those 16 cases, 9 (56%) had a history of recurrent EHI events, which were found to be frequent when compared with the 14 (37.8%) cases with recurrent EHI among the remaining 37 cases without pathogenic variants. Two *RYR1* variants were identified in two of our EHI cases, both presenting with muscle symptoms and increased creatine kinase (Table S4). One case with the p.Arg2435Leu variant, which is known to cause MH [26], did not have any family or personal history of MHS. The other case that showed positive CHCT results carried the p.Val4828Met variant associated with MH and core myopathies [26,27]. Our remaining three CHCT-positive EHI cases were negative for pathogenic and or potentially pathogenic variants of all MH candidate genes. All cases in the study cohort were also negative for variants in the genes *ASPH* and *TRPV1*, both recently implicated in EHI [11,12].

Four EHI cases had variants in dominantly inherited cardiomyopathy genes, including two with *MYH7* variants that affected highly conserved amino acids within myosin S2 and light meromyosin domains of the beta myosin (Supplemental Figure S1). All four cases had experienced collapse or syncopal events (Table S4). However, only one case with the *TRPM4* p.Arg905Trp variant was evaluated for cardiac function with normal results. The remaining cases were carriers of pathogenic variants in genes implicated in autosomal recessive myopathies and mitochondrial disorders (Table 3). The majority of these carriers presented with signs of CNS that included collapse, loss of consciousness, altered mental status, or combinations of those (Table S4).

The genes identified in this study are associated with distinct AD and AR disorders with highly variable clinical presentations and phenotypes. This prompted us to examine clinical phenotypes overrepresented in these genetic disorders using gene-set-based phenotype enrichment analysis using the Human Phenotype Ontology (HPO) database, which contains all of the human-disease-gene-associated phenotypes known to date [28]. Results show a number of clinical phenotypes that were significantly enriched in the EHI study cohort (Supplemental Table S5). Rhabdomyolysis, metabolic acidosis, myalgia, lethargy, and signs of CNS dysfunction (reduced consciousness and confusion) are known clinical phenotypes of EHI [1,2,3]. However, some of the phenotypes—such as abnormal mitochondrial metabolism, arrythmia, cardiomyopathy, and abnormal heart morphology—are rarely documented in EHI cases. Importantly, these phenotypes, especially cardiac phenotypes, were shared by 8–10 of the 14 genes identified in this study. In addition to these shared features, proteins encoded by 12 genes show direct functional and/or physical interactions (Supplemental Figure S2).

### 2.4. Validation of TRPM4 Variant Pathogenicity in EHI

Among the 12 genes with novel associations with EHI, *TRPM4* was of particular interest. This gene encodes a non-selective, Ca^2+^-impermeable cation channel, TRPM4, that belongs to the heat-activated TRP channel family [29,30]. This channel is emerging as a thermal-sensitive channel that may have a potential role in thermoregulation, a topic highly relevant to our study population [31]. Although variants in *TRPM4* have been associated with arrythmias [18,32], our case, with the p.Arg905Trp variant, demonstrated recurrent EHI that was found to be complicated by syncope and which was diagnosed as MH susceptible (Table S4), with no variants in MH candidate genes [9,10,11,12]. While pathogenic variants in *TRPM4* have been associated with cardiac phenotypes, the subject’s electrocardiogram and echocardiogram were normal following the event. TRPM4 is expressed in skeletal muscle (Supplemental Figure S3), but its role is unclear [33]. Based on the clinical characteristics of the case, we hypothesized that p.Arg905Trp might affect TRPM4 channel function with respect to Ca^2+^ sensitivity, voltage activation, or temperature modulation.

#### 2.4.1. Analysis of the TRPM4 Variant Impact on Channel Structures

**Figure 1:**
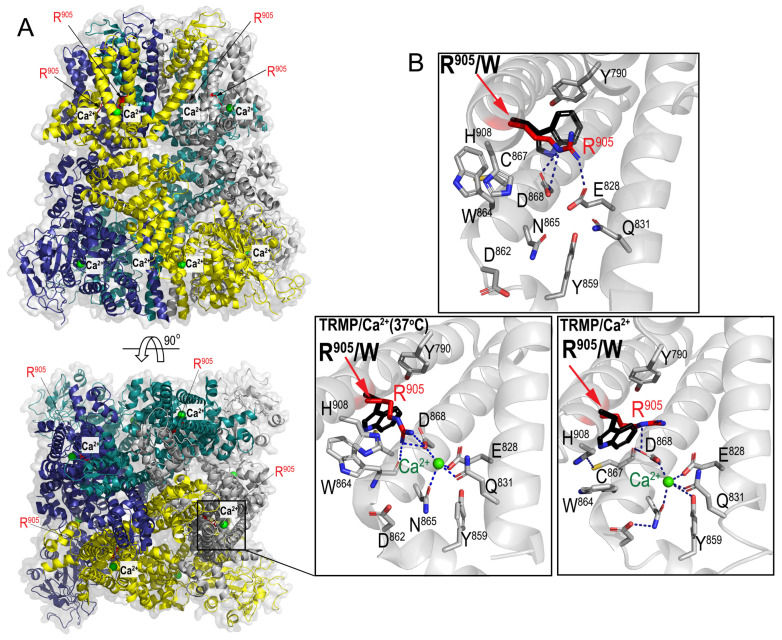
Predictions of the p.Arg905Trp variant impact in the structural assembly and function of TRPM4. (**A**) The ribbon structure of the TRPM4 tetramer in the Ca^2+^-bound state at 37 °C (PDB: 9B8W [31]) with tetramer protomers colored cyan, grey, yellow and blue. TRPM4 is tetramer, Ca^2+^ molecules are shown as green spheres, and Arg905 (R905) residues are displayed in red. (**B**) Enhanced view of the network of interactions involving R905 in TRPM4 without Ca^2+^ (top panel, as in PDB: 6BQR [30]) and with Ca^2 +^ (bottom panel) at 37 °C (PDB: 9B8W) and in cryo-conditions (PDB: 6BQV). Hydrogen bonds formed by Arg905 are shown as blue dashes. The Trp residue modeled to replace R905 (red sticks) is shown in black.

The p.Arg905Trp variant resides in the fourth transmembrane domain of the channel and changes a highly conserved amino acid (Supplemental Figure S4). We analyzed the potential changes to overall protein assembly and function caused by the variant using available crystal structures of human TRPM4 in Ca^2+^-free and Ca^2+^-bound states [30,31]. As shown in Figure 1, Arg905 is directly involved in the network of interactions stabilizing both the apo- and Ca^2+^-bound states of TRPM4. In the unbound, closed conformation (Figure 1B, top panel), Arg905 coordinates the Glu828 and Asp868 residues of the primary Ca^2+^ binding site, which is located at the interface between the transmembrane domain (TMD) and the intracellular domain (ICD) of TRPM4. This site has been resolved in the TRPM4 structures determined under cryogenic conditions and at 37 °C [30,31]. Upon Ca^2+^ binding, the side chains of Glu828 and Asp868 are released from interactions with Arg905 to facilitate direct coordination with Ca^2+^ as part of this Ca^2+^-binding pocket (Figure 1B, bottom panel). We speculate that the replacement of Arg905 by a bulky hydrophobic Trp residue will disrupt the network of interactions provided by the guanidinium group of Arg905. In addition, a Trp residue may donate a hydrogen to the network of hydrogen bonds and partially substitute for the guanidinium group of Arg905. These results show that the *TRPM4* p.Arg905Trp variant will likely impair the channel function by interference with a network interaction within the primary Ca^2+^ binding site at the TMD/ICD interface. A recent study of TRPM4 at 37 °C identified an additional Ca^2+^ binding site named Ca^2+^_warm_, located at the interface between the MHR1/2 and MHR3/4 domains [31]. This newly described site is situated around residues 377–413, where the glutamic acid at position 396 (Glu396) plays an important role in temperature-induced conformational transitions. Our structural analyses of TRPM4 at 37° indicate that Glu396 and Arg905 are within separate subunits, relative to the Ca^2+^ binding sites, where each is coordinated by different regions of TRPM4 [30,31]. As these two Ca^2+^ binding sites are thought to be not allosterically coupled, we speculate that Arg905Trp likely has no effect on the function of Glu396 in coordinating the Ca^2+^_warm_ site. Based on overall structural data, we suggest these sites function independently, with the R905W variant being detrimental to the primary Ca^2+^ binding site, while leaving the newly proposed one with the Glu396 unaffected.

#### 2.4.2. Functional Study of TRPM4 Variant in Excised Patches

**Figure 2:**
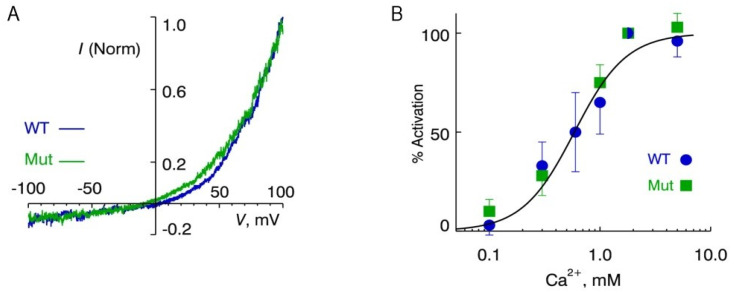
Electrophysiology of the WT- and R905W TRPM4 channels. (**A**) Current–voltage relationship of WT and R905W (Mut) TRPM4 channels in excised patches. These were recorded during a voltage ramp from −100 to +100 mV (0.4 V/s) after patch excision into a bathing solution containing 1.8 mM Ca^2+^. Record amplitudes are normalized to the value at +100 mV. (**B**) Sensitivity of WT and R905W channels to Ca^2+^ in the continued presence of 8 µM PIP_2_. An amount of 1.8 mM Ca^2+^ was taken as reference. Each point represents the normalized mean ± SEM of 2–5 determinations. The solid line is a fit of the data to a Hill equation with *EC*_50_ activation at 0.59 mM and *n* = 1.85.

TRPM4 is a Ca^2+^- and voltage-activated channel modulated by phosphatidylinositol-4,5-bisphosphate (PIP_2_) and heat [29,30,31]. To test our hypothesis and assess the functional consequences of the *TRPM4* p.Arg905Trp (R905W) variant, we evaluated TRPM4 currents from transiently expressed, fluorescently labeled WT and R905W (Mut) channels in HEK cells. The channels were recorded in excised patches in order to control exposure to activating Ca^2+^ and PIP_2_ (Supplemental Figure S5). TRPM4 activation by Ca^2+^ and PIP_2_ were similar between WT and R905W channels. As shown in Figure 2, we analyzed the current properties of the channels in response to changes in membrane voltage and an increase in intracellular Ca^2+^. The *I–V* curves for both WT and R905W channels show pronounced outward rectification. WT and R905W channels demonstrate similar responses to voltage change from −100 to +100 mV (Figure 2A). The steady-state Ca^2+^ dependence of channel activation was also similar in excised patches from WT and R905W channels (Figure 2B, Supplemental Figure S5A,B), with an EC_50_ of 0.59 mM. Taken together, these results show that R905W did not affect the Ca^2+^- and voltage-dependent functional properties of the TRPM4 channel.

**Figure 3:**
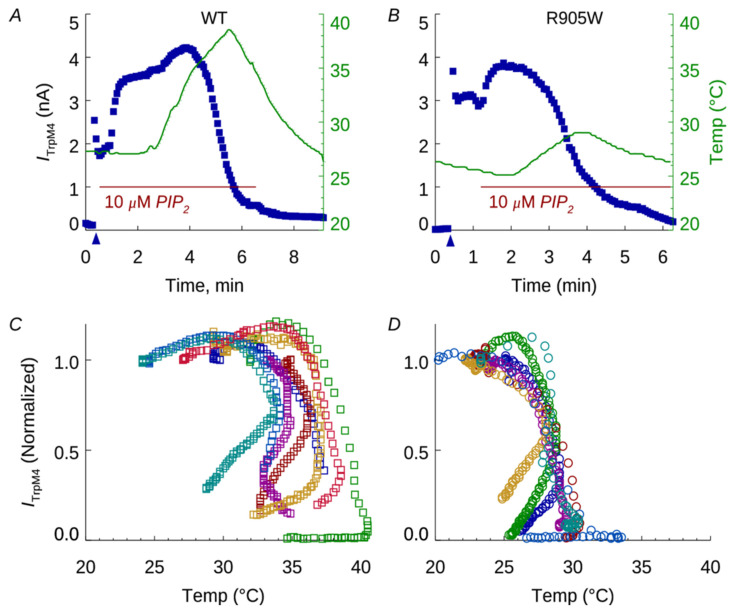
Temperature dependence of TRPM4 channel currents. (**A**,**B**) WT (**A**) and R905W mutant (**B**) channel currents were recorded from excised patches in the continuous presence of 1.0 mM Ca^2+^ and 10 µM PIP_2_, as indicated. Elevation of bath temperature (right-hand axis, solid green line) resulted in rapid decline of current. Up arrows indicate time of patch excision. (**C**,**D**) TRPM4 currents plotted against temperature show that WT currents (**C**) declined rapidly around 33–37 °C, while those of R905W mutants (**D**) declined by 25–30 °C. Each symbol color represents a different excised patch.

We further examined the activities of the WT and the R905W TRPM4 channels in response to an increase in temperature with a ramp of 25–37 °C (Figure 3). In the presence of activating Ca^2+^ (1.0 mM) and 10 µM PIP_2_, the elevation of bath temperature resulted in an early increase, and then a rapid decrease, in current. In the WT cells (Figure 3A), current suppression commenced at or above *~*33 °C, while in the R905W channels current suppression was below 30 °C (Figure 3B). Subsequent lowering of the temperature did not result in the re-activation or recovery of current, despite the continued presence of Ca^2+^ and PIP_2_. When the time course of the TRPM4 current was plotted against temperature, the current in WT patches (Figure 3C) was suppressed at temperatures significantly higher than the desensitization of the R905W channels (Figure 3D). The mean temperature for 50% suppression was 35.6 ± 0.73 °C (n = 9, mean ± SEM) in patches from WT cells and 28.6 ± 0.16 °C in patches from R905W cells (n = 8; *p* < 2.5 × 10^−7^). These results demonstrate the pronounced sensitivity of the R905W channel to temperature elevation, compared with the WT channel, in the excised patches. Following heat-induced activation, the R905W channel current showed a temperature-dependent inactivation at a lower temperature than that of the WT channel. This premature inactivation of the R905W TRPM4 channel is likely to affect the membrane depolarization that might lead to Ca^2+^ dysregulation in muscle cells.

## 3. Discussion

Despite ongoing studies to elucidate EHI etiology and risk factors, research investigating the role of genetics in the development of EHI remains in its infancy. Here, we studied genetic factors contributing to individual variability in the susceptibility to EHI using the WES approach in a cohort of 53 independent EHI cases. We identified clinically significant pathogenic and potentially pathogenic variants in 16 cases (30.2%) of the study cohort. Variants were found in 14 genes, including 12 genes reported for the first time in association with EHI. We also found that recurrent EHI events were more frequent (9 of 16) among cases with pathogenic variants compared with cases (14 of 37) without pathogenic variants, suggesting a contribution of genetic factors in EHI recurrence. In genetic studies of exertional rhabdomyolysis frequently associated with EHI, the recurrence of events can indicate underlying metabolic and other muscle disorders [20,21].

Previously, EHI has been associated with variants in *RYR1* [6,7,8,9]. We identified two, pathogenic and potentially pathogenic, *RYR1* variants in two EHI cases. While one of these cases was evaluated and diagnosed with MH, the other one had no personal and/or family history of MH. Although uncommon, *RYR1* pathogenic variants in cases without history of MH have been reported [9]. These cases are likely due to the low penetrance of MH-causative pathogenic variants and/or the lack of history of exposure to triggering anesthetics. The negative results of MH candidate genes in three other CHCT positive EHI cases could be explained by low sensitivity of CHCT and/or other unknown genetic factors. It is worth noting that CHCT are highly sensitive to abnormal myoplasmic Ca^2+^ regulation. While there is an obvious difference in clinical presentation and disease mechanisms between MH and EHI, a feature common to both conditions is that they are triggered by environmental factors such as heat and exercise, which contribute to elevated myoplasmic Ca^2+^ concentration. Overall, the prevalence of *RYR1* pathogenic variants was found to be low, as shown here and by other studies [7,8], thus demonstrating further genetic heterogeneity in EHI.

Significant findings from this study were the variants in autosomal dominant cardiomyopathy/arrhythmia (*MYH7*, *RYR2* and *TRPM4*) genes. The *RYR2* p.Arg485Gln variant has been described in independent cases with left-ventricular non-compaction [34,35]. Two variants, p.Glu949Val and p.Asp1339Gly, were identified in *MYH7*, one of the major genes for hypertrophic cardiomyopathy (HCM) [16]. These variants change highly conserved amino acids within protein domains enriched with HCM variants [36]. While this study reports the *MYH7* p.Asp1339Gly, others have described p. Glu949Val in patients with HCM [24,37]. Although these *RYR2* and *MYH7* variants are classified as VUS, based on their association with cardiomyopathies and evidence of high pathogenic scores, we suggest that these variants may negatively impact cardiac output during vigorous exercise and might contribute to the development of EHI. The *TRPM4* p.Arg905Trp variant found in our study case, with recurrent EHI and MH diagnosis, highlights the pleiotropic effect of this gene, which is associated with arrhythmic syndromes and erythrokeratodermia [18,32,38]. However, in contrast with known disease-associated *TRPM4* variants, the p.Arg905Trp did not change Ca^2+^ sensitivity, Ca^2+^, or voltage dependence of channel activation (Figure 2), which was unexpected given the MH phenotype of the case. Remarkably, the p.Arg905Trp variant affected thermal sensitivity of the TRPM4 channel and inactivated its function at lower temperatures in excised patches compared with the wild type (Figure 3). It is conceivable that heat-generating muscle contraction during exertion will impair the channel function in a temperature-dependent manner, leading to the development of the recurrent EHI observed in our case. As predicted by our structural analysis, a potent contributor to this “heat sensitive” phenotype is the bulkiness of Trp residue at the amino acid position 905 that directly impacts the coordination of a Ca^2+^ ion in its binding site (Figure 1) [30,31]. Interestingly, the Arg905Trp has no allosteric interaction with the recently proposed new Ca^2+^ binding site, where Glu396 residue plays a key role in the temperature-driven conformational transitions of TRPM4 [31]. Nevertheless, our overall data suggest the independent function of these sites in the thermal sensitivity of TRPM4, where the R905W variant is detrimental to the primary Ca^2+^ binding site and does not affect, newly described sites including Glu396. We speculate that temperature-sensitive inactivation of the R905W TRPM4 channel will likely affect membrane potential, leading to the Ca^2+^ dysregulation in muscle cells, which may in part explain the MH diagnosis of the case. However, the role of TRPM4 in muscle cells and the effects of volatile anesthetics on its function remain to be determined.

We additionally identified a novel association between EHI and genes implicated in cellular energy production. Although genes associated with similar functions have been previously investigated in EHI [8], the study was limited in terms of the number of screened genes. Here, we confirm and expand the role of OXPHOS by detecting pathogenic variants in genes for mitochondria, electron transport chains (*C1QBP*, *NDUFA6*, *NDUFAF5* and *NDUFS7*), fatty-acid oxidation (*ACADVL*, *HADHB*, *ETFB*), phosphate (*PPA2*) and muscle Ca^2+^, and glycogen (*CAPN3*, *PFKM*) metabolism [20,23,39]. Prominent features of fatty-acid oxidation deficiencies are multiorgan dysfunction and exercise intolerance [20]. Patients with electron transport chain defects express CNS dysfunction and metabolic acidosis [23]. Of those variants identified in EHI cases, the *CAPN3* p.Asp753Asn has been reported in patients with hyperCKemia [40], and the p.Leu202Pro has been reported in a heterozygous state in two independent patients with residual enzyme activities [41,42]. The *NDUFA6* p.Ile94LysfsTer44 variant has been reported in a patient with recurrent exertional rhabdomyolysis [43], and the *C1QBP* p.Thr40AsnfsTer45 and *PPA2* p.Glu172Lys variants have been reported in autosomal recessive mitochondrial cardiomyopathies with heart failure and sudden cardiac death [44,45]. It is important to note that our cases were heterozygous carriers of variants associated with autosomal recessive disorders. However, variants identified in EHI cases were pathogenic variants, including loss-of-function variants with known disruptive effects as shown by the literature data. We propose that these pathogenic variants will likely affect ATP production in striated muscles, thus impacting OXPHOS during energy-demanding exertion.

In the current study, about 1 in 3 cases carried pathogenic or potentially pathogenic variants in 1 of 14 genes identified. These genes are associated with distinct genetic disorders, with highly variable clinical presentations and symptomology. Despite this heterogeneity, abnormal mitochondrial metabolism, arrythmia, and cardiomyopathy were the most common clinical phenotypes, shared by 8–10 genes. Moreover, the majority of genes also show direct interactions supporting their functional relationship and potential role in EHI. The results and genetic risk factors identified in this study should be considered in future clinical genetic testing and clinical studies of EHI.

Study limitations: The study approach was limited to coding regions of the genome. Variants may exist in gene regulatory regions and/or deep within introns due to the incomplete sequence depth of these regions by WES. In addition, phenotype–genotype correlation analyses of variants, especially in genes with a new association with EHI, were not studied in detail. However, this is partly due to the nature of EHI clinical diagnoses, which often do not include a cardiac workup. The study sample size was modest, which is important for clinically and genetically heterogenous conditions.

## 4. Materials and Methods

This study was approved by the Institutional Review Board of the Uniformed Services University (USU). Informed consent was obtained from each participant before enrollment in the study.

### 4.1. Study Population

Individuals diagnosed with complex EHI (recurrent or assessed to be clinically severe) were referred to the Human Performance Laboratory of the Consortium for Health and Military Performance (CHAMP), USU, for further evaluation in order to facilitate their return to activity/duty recommendations. Among those, 53 (51 service members, 1 veteran, and 1 college athlete) volunteered to enroll in this genetic study. Six patients were additionally evaluated for MH using caffeine–halothane contracture tests (CHCT) [46]. Clinical characteristics and laboratory test results obtained from referring providers and/or from electronic health records were examined. Laboratory test results include alanine amino transferase (ALT), aspartate amino transferase (AST), blood urea nitrogen (BUN), creatinine (Cr), serum creatine kinase (CK), and sodium and potassium. Self-reported questionnaires about demographics, physical characteristics, drug and supplement use, and medical and family histories were also examined. Medical records and self-reported questionnaires were examined for environmental factors at the time of the events in order to include details of exercise activity and location and date of EHI events.

### 4.2. Whole-Exome Sequencing (WES) and Variant Analyses

WES was performed using genomic DNA. Details of sample preparation, WES, and raw data analysis are provided in the Supplemental Methods. Nonsynonymous, splice, stop gain, and stop loss variants were annotated with SnpEff (v5.0, https://pcingola.github.io/SnpEff, the last accessed on 28 February 2024) and prioritized with minor allele frequency (MAF) filtering criteria of ≤0.1% for autosomal recessive (AR) and ≤0.01% for autosomal dominant (AD) disorders [19]. MAFs were identified using the population maximum frequencies of variants in the continental populations from the Genome Aggregation Database, composed of >464,000 exomes and >63,000 genomes (gnomAD, v4.0; https://gnomad.broadinstitute.org, the last accessed on 2 August 2024). Filtered variants were further analyzed by querying ClinVar, which provides classification of variants according to the American College of Medical Genetics and Genomics (ACMG) guidelines [25,47]. Rare variants, classified by independent submitters as benign or likely benign in ClinVar, were excluded from further analysis. Rare variants, classified as pathogenic (P), likely pathogenic (LP), variant of uncertain significance (VUS), and variants with no annotation (NA) in ClinVar, were analyzed in detail. Two widely used in-silico algorithms—Combined Annotation Dependent Depletion (CADD) and Rare Exome Variant Ensemble Learner (REVEL)—were used to predict the pathogenic effects of VUS and NA variants [48,49]. We used a CADD score of ≥20 as the initial cutoff for nonsynonymous variants [48]. More stringent criteria, with a CADD score ≥28 and REVEL scores of 0.77–0.93, were used as supporting evidence of variant pathogenicity for nonsynonymous variants within the ACMG criteria [50]. Clinically relevant novel variants were submitted to ClinVar (submission ID: SUB14704056; https://www.ncbi.nlm.nih.gov/clinvar/, submitted on 3 September 2024). Genes with P, LP, and VUS and with high pathogenicity metascores were investigated for gene set phenotype enrichment and protein interaction using STRING (v12.0, https://string-db.org, assessed on 14 March 2024) portal [28].

### 4.3. Structural Prediction and Functional Study of TRPM4 p.Arg905Trp Variant

We performed protein structural and functional studies to determine pathogenicity of the *TRPM4* p.Arg905Trp variant found in this study. *TRPM4* encodes the transient receptor potential melastatin 4 (TRPM4) channel, which belongs to the heat-activated TRP channel family [29,30]. A molecular model of TRPM4 with the p.Arg905Trp (R905W) variant was generated using available crystal structures of human TRPM4 (hTRPM4) in Ca^2+^-free and Ca^2+^-bound states [30,31]. Structure-based alignment and variants were generated using COOT [51]. Figures were made with Pymol Molecular graphics (http://pymol.org, last retrieved on 27 September 2024).

The wild-type (WT) hTRPM4 plasmid (plasmid # 25903, Addgene, Watertown, MA, USA) was kindly provided by Dr. C. Montell [29]. The R905W variant was introduced using site-directed mutagenesis (see Supplemental Methods). The WT and the R905W TRPM4 channels were inserted into a pCMV6-AC-GFP vector (Origene, Rockville, MD, USA). HEK293 cells were transfected with WT or R905W TRPM4 constructs. Protein expression levels were analyzed by Western blot and detected with antiTRPM4 antibody (ABN418, MilliporeSigma, Rockville, MD, USA). Experimental conditions and details of electrophysiological studies are given in the Supplemental Methods. TRPM4 channel currents were recorded from inside-out patches excised from cells after the formation of a giga-seal. Bath temperature was controlled by an in-line heater plus controller (Warner Instruments TC324B, Harvard Apparatus, Holliston, MA, USA). The temperature was monitored by a meter with voltage-proportional output (Omega) and a thermocouple mini-probe placed near the pipette. TRPM4 currents were recorded during voltage ramps from +100 to −100 mV (0.4 V/s) every 4 s. The time course of changes of current magnitude (*I*_TRPM4_) were calculated as *I*_+100_ − *I*_−100_ for each ramp.

### 4.4. Data and Statistical Analysis

Data analysis was conducted using the Statistical Package for the Social Sciences (SPSS, version 25) software program. Based on data distribution, data were presented as median (range) or as mean ± standard deviation. The Student’s *t*-test was used for between-group comparisons, and statistical significance was assumed as *p* < 0.05. Apparent temperature was determined using collected data in locations and on the date of an EHI event and on the daily average universal thermal climate index of the day that the event occurred. This index was estimated using meteorological data available in the ERA5-thermal dataset [52,53] and at the geographic centroid of the installation at which the event occurred [54]. Patch clamp data were analyzed using PClamp 10 software (version 10.6, Molecular Devices, San Jose, CA, USA), and statistical analysis was performed with Interactive Data Language (IDL) software (IDL version 8.9, Harris Geospatial Solutions, Boulder, CO, USA).

## 5. Conclusions

In this study, we performed exome sequencing in a cohort of 53 EHI cases. WES, followed by various analytical approaches, identified clinically significant pathogenic and potentially pathogenic variants in 30.2% of this study cohort. We found that more than half of cases with pathogenic variants had recurrent EHI events. Genetic results demonstrate novel associations between EHI and 12 genes largely related to cardiomyopathies and OXPHOS deficiencies. This suggests that some genetic risk factors may affect cardiac function and energy production during vigorous exercise and might increase susceptibility to EHI. Supporting structural and functional studies of the *TRPM4* p.Arg905Trp variant demonstrate that this variant impairs the thermal sensitivity of the TRPM4 channel, a potentially new molecular mechanism of EHI. Our findings expand the genetic heterogeneity of EHI and provide further research avenues into the genetics and pathogenic mechanisms of EHI. The results from this study may inform clinical and genetic studies of individuals with EHI.

## Figures and Tables

**Table 1:** Demographic and clinical characteristics of the study cohort.

Characteristics (*n* = 53, Except as Indicated)	Reported	Normal Range
**Sex:** n (%)		
Male	46 (86.8)	
Female	7 (13.2)	
**Age:** median (range)	27 (19–41)	
**Peak core temperature, (°C):** median (range)	41.1 (38.5–43.0)	37 (36.1–37.2)
**Number of events:** n (%)		
Single	30 (56.6)	
Recurrent	23 (43.4)	
**Clinical signs and symptoms (*n* = 47):** n (%)		
Collapse/syncope	27 (57.4)	
Altered mental status	24 (48.9)	
Muscle cramping/pain	20 (42.5)	
Muscle weakness/fatigue	19 (40.4)	
**Laboratory test results (*n* = 37–44):** median (range)		
Alanine amino transferase (IU/L)	87 (22–700)	7–56
Aspartate amino transferase (IU/L)	129 (35–700)	8–33
Blood urea nitrogen (mg/dL)	19 (12–30)	6–24
Creatinine (mg/dL)	1.6 (1.1–2.5)	0.7–1.3
Peak creatine kinase (IU/L)	1766 (189–42,299)	30–200
Potassium (mEq/L)	4.4 (3.4–5.5)	3.5–5.5
Sodium (mEq/L)	142 (135–155)	135–145
**Personal or family history:** n (%)		
Malignant hyperthermia susceptible	4 (7.5)	
Sickle cell trait	2 (3.8)	
Family history of hypertension	2 (3.8)	
**Type of exercise activity on the day of event (*n* = 34):** n (%)		
Running 6.5–14.5 km	16 (47.1)	
March, hike, and or run with weight, endurance training	13 (38.2)	
Other (training run, task-force training)	5 (14.7)	
**Apparent temperature † (*n* = 27):** °C (mean ± SD)	25 ± 4	

† the measure is based on the universal thermal climate index.

**Table 2:** Summary of whole-exome sequencing results.

Associated Conditions	Number of Genes	Number of Variants	Classifications per ClinVar †		
			P/LP	VUS	NA
Muscle disorders of metabolism, contraction and calcium dysregulation	16	27	2	15	10
Mitochondrial oxidative phosphorylation deficiencies	28	30	4	17	9
Cardiomyopathies, arrhythmias	22	40	0	31	9
Initial classification of variants	66	97	6	63	28
Classification of variants after assessments	66	97	8	89	—

† P = pathogenic, LP = likely pathogenic, VUS = variant of uncertain significance, NA = variants with no annotation.

**Table 3:** Pathogenic and potentially pathogenic variants found in EHI cases.

Gene Symbol, Amino Acid Variant	ClinVar †	Metascores		ACMG	Associated Disease, Inheritance
		REVEL	CADD		
Genes previously associated with EHI					
*ACADVL:* p.Leu202Pro	LP	0.95	32	LP	Very-long-chain acyl-CoA dehydrogenase deficiency, AR
*RYR1:* p.Arg2435Leu	LP	0.94	32	LP	Malignant hyperthermia, AD; core myopathy, AD, AR
p.Val4842Met	VUS	0.93	29	VUS	
Genes reported for the first time in association with EHI					
*C1QBP:* p.Thr40AsnfsTer45	NA	NR	NR	LP	Combined OXPHOS deficiency, AR
*CAPN3:* p.Asp753Asn	VUS	0.65	25	VUS	Limb-girdle muscular dystrophy, AR,
*ETFB:* p.Pro93GlnfsTer45	NA	NR	NR	LP	Mitochondrial glutaric aciduria II, AR
*HADHB:* p.Gly301Ser	VUS	0.98	29	VUS	Mitochondrial trifunctional protein deficiency, AR
*MYH7: * p.Glu949Val	VUS	0.91	28	VUS	Hypertrophic cardiomyopathy, AD; myosin myopathy, AD, AR
p.Asp1339Gly	NA	0.95	29	VUS	
*NDUFAF5:* p.Ser137GlnfsTer5	LP	NR	26	LP	Mitochondrial complex I deficiency, AR
*NDUFA6:* p.Ile94LysfsTer44	LP	NR	29	LP	Mitochondrial complex I deficiency, AR
*NDUFS7:* p.Arg111Cys	NA	0.85	30	VUS	Mitochondrial complex I deficiency, AR
*PPA2:* p.Glu172Lys	P	0.51	30	P	Mitochondrial inorganic pyrophosphate deficiency, AR
*PFKM:* p.Arg246Ter	P	NR	40	P	Glycogen storage disease VII, AR
*RYR2:* p.Arg485Gln	VUS	0.79	31	VUS	Catecholaminergic polymorphic ventricular tachycardia, AD
*TRPM4:* p.Arg905Trp	VUS	0.84	25	LP	Cardiac conduction defects, arrhythmia, AD

† P = pathogenic, LP = likely pathogenic, VUS = variant of uncertain significance, NA = variants with no annotation, AD = autosomal dominant, AR = autosomal recessive.

## Data Availability

All relevant data are within the manuscript. Additional supporting data are included in Supplemental Materials. Further inquiries can be directed to the corresponding author.

## Supplementary Materials

The following supporting information can be downloaded at: https://www.mdpi.com/article/10.3390/ijms252011269/s1.
