# Supplementary material for: Genetics of Exertional Heat Illness: Revealing New Associations and Expanding Heterogeneity
Source: Int J Mol Sci. 2024 Oct 19;25(20):11269. doi: 10.3390/ijms252011269 (PMC11508780; doi:10.3390/ijms252011269)
Supplement: Supplementary file 1 [file ijms-25-11269-s001.zip › Supplemental_Materials.pdf]

Genetics of Exertional Heat Illness:

Revealing New Associations and Expanding Heterogeneity

Nyamkhishig Sambuughin, Ognoon Mungunsukh, Michael G. Klein, Ming Qiang Ren, Peter Bedocs, Josh B. Kazman, Kristen Cofer, Liam P. Friel, Beth McNally, Kyung Kwon, Mark C. Haigney, Jeffrey C. Leggit, Marzena Pazgier, Patricia A. Deuster, Francis G. O'Connor

SUPPLEMENTAL MATERIAL

Supplemental Methods

Whole-exome sequencing

Genomic DNA was extracted from peripheral blood using the QIAamp DNA blood mini kit (Qiagen) following the manufacturer's instructions. Genomic DNA quantitation was performed using a Qubit® dsDNA HS Assay Kit (molecular probes) and measured by an Invitrogen™ Qubit™ 4 fluorometer. DNA was subjected to whole-exome sequencing (WES) commercially (Azenta Life Sciences). Variant calling and annotations have been reported in detail previously [1]. Briefly, raw sequencing reads were aligned to the reference genome (NCBI GRCh38) using BWA-MEM. VCF files were used for annotation and further analysis.

Cell culture, mutagenesis, and transfection

The p.Arg905Trp (R905W) variant was introduced into pcDNA3.1 plasmid containing wild-type (WT) human TRPM4 using site-directed mutagenesis with the Quick-Change Site Directed Mutagenesis kit according to the manufacturer's protocol (Agilent Technologies, Santa Clara, CA). The integrity of all plasmids was confirmed by DNA sequencing. The WT- and R905W-TRPM4 were inserted into a pCMV6-AC-GFP vector (Origene) with C-terminal tagged *Aequorea coerulescens* (AC) green fluorescent protein (GFP). Cell culture and cell transfections were performed as described previously [2]. Cells were grown in 35 mm culture dishes until 70–80% confluence was reached and transfected with plasmids. Cells were transferred to coverslips the next day and used for experimentation at 48 h after transfection.

TRPM4 expression

TRPM4 expression profiles were obtained from the genotype-tissue expression (GTEx) data, a comprehensive public resource to study tissue-specific gene expression (www.gtexportal.org). Levels of WT- and R905W-TRPM4 proteins transiently expressed in HEK cells were analyzed using Western blot [3]. In short, cells were collected and lysed in protein lysis buffer on ice for an hour. Cell lysates were cleared with centrifugation, and supernatants were used as input for total expression of TRPM4. Proteins were detected with antiTRPM4 (ABN418, Millipore) and antiActin (Santa-Cruz) antibodies.

Electrophysiology

HEK-293 cells expressing WT-TRPM4 or R905W-TRPM4 were identified via epifluorescence of co-expressed GFP. Currents were recorded from inside-out patches excised from cells after formation of a giga-seal. An Axopatch 200B amplifier plus Digidata 1440a interface under control of PClamp 10 software (molecular devices) was used to monitor current and provide voltage

commands. Currents were digitized at 10 kHz and filtered at 5 kHz. The extracellular solution contained (mM): NaCl 140, KCl 5.4, HEPES 10, $CaCl_2$ 1.8, $MgCl_2$ 1, glucose 10, pH 7.4. The pipette solution and solutions for patch excision were similar (except for glucose) in order to prolong cell viability and eliminate junction potentials. Patch pipettes were pulled (Sutter Instruments) and fire-polished (Narashige) to a resistance of 1–3 MΩ. Ca^{2+} levels below 0.6 mM in the patch experiments were set by total Ca^{2+} plus Br_2-BAPTA (2 mM) according to MaxChelator software (https://somapp.ucdmc.ucdavis.edu/pharmacology /bers/maxchelator). Exogenous diC8 phosphatidylinositol 4,5-bisphosphate (PIP_2; 8–10 µM; Echelon Biosciences) was added as indicated. Solutions were applied via gravity flow from different reservoirs through a manifold and removed by aspiration.

Supplemental Results

Genetic results

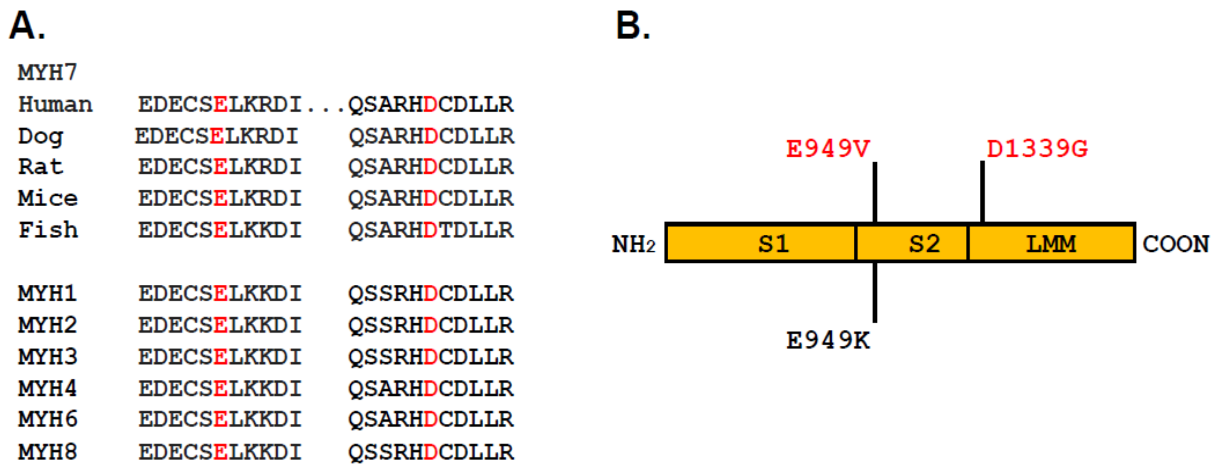

Figure S1. Amino acid alignment and variant locations within MYH7. **A.** Glutamic acid (E) at 949 and aspartic acid (D) at 1339 positions are highly conserved within MYH7 of different species and between various subtypes of human myosins. **B.** The p.Glu949Val and p.Asp1339Gly variants are located in S2 and in light meromyosin domains of the protein, respectively. The pathogenic variant at the same position, p.Glu949Lys, is causative of hypertrophic cardiomyopathy [4,5].

Table S4. Clinical features of EHI cases with pathogenic and potentially pathogenic variants

**ID#**	**Sex, Age**	**EHI events**	**Peak core Temp: °C**	**Medical record data**	**Peak CK† (UI/L)**	**Genetic finding††**
HP019	M,25	1	40.4	Collapsed, unresponsive	400	*ACADVL:*p.Leu202Pro
HP013	M,19	1	NR*	Muscle pain, cramps, hematuria	5,500	*RYR1*: p.Arg2435Leu
HP415	M, 31	2	41.8	Muscle cramps, pain, fatigue, acute kidney injury, MH susceptible by positive CHCT	15,000	*RYR1*: p.Val4842Met
HP001	F, 20	1	40.5	No collapse, alert	NA	*C1QBP:* p.Thr40AsnfsTer45
HP036	F, 20	1	42.2	Collapsed, altered mental status	425	*CAPN3:* p.Asp753Asn
USU75	M, 29	2	41.7	Collapsed, loss of consciousness, confused, muscle weakness	519	*ETFB:* p.Pro93GlnfsTer45
HP052	M, 23	2	40.5	Alert, diaphoretic, cardiac examination and EKG normal	827	*HADHB:*p.Gly301Ser
HP058	M, 22	2	41.7	Collapsed, loss of consciousness, altered mental status, nausea, acute kidney injury, heart palpitations	23,970	*MYH7*: p.Glu949Val
HP051	M, 25	1	41.1	Collapsed, loss of consciousness, shortness of breath, muscle pain	1,104	*MYH7*: p.Asp1339Gly
HP055	M, 29	2	41.0	No collapse, altered mental status	297	*NDUFAF5:* p.Ser137GlnfsTer5
HP044	M, 29	2	42.4	Collapsed, altered mental status, muscle weakness	6,173	*NDUFA6:* p.Ile94LysfsTer44
HP012	M, 21	1	41.4	No collapse, altered mental status.	9,073	*NDUFS7*: p.Arg111Cys
HP060	M, 35	1	41.05	Collapsed, altered mental status, muscle pain, myoglobinuria	>2,000	*PPA2*: p.Gln172Lys
HP014	M, 26	2	40.6	NA	NA	*PFKM:* p.Arg246Ter
USU30	M, 25	2	41.7	Collapsed, loss of consciousness, altered mental status, vomiting	282	*RYR2:* p.Arg485Gln
HP176	M, 21	2	NA	Syncope, muscle cramps, shortness of breath, chest pain. Cardiac examination, EKG and ECHO were normal. MH susceptible by positive CHCT	6,068	*TRPM4*: p.Arg905Trp

† - Creatine Kinase; †† - All variants were in heterozygous states; NA –not available

Table S5. Phenotype enrichment analyses of genes associated with EHI

HPO Term #	HPO Term	Gene #	Strength†	Matching Genes in This Study
0002913	Myoglobinuria	4	2.37	*HADHB, RYR1, ACADVL, PFKM*
0003201	Rhabdomyolysis	4	2.17	*HADHB, ETFB, RYR1, ACADVL*
0008316	Abnormal mitochondria	4	2.16	*NDUFS7, MYH7, NDUFAF5, NDUFA6*
0000114	Proximal tubulopathy	4	2.1	*NDUFS7, ETFB, NDUFAF5, NDUFA6*
0003326	Myalgia	6	1.82	*HADHB, ETFB, MYH7, RYR1, CAPN3, ACADVL*
0001942	Metabolic acidosis	7	1.81	*C1QBP, NDUFS7, PPA2, ETFB, RYR1, ACADVL, NDUFA6*
0003287	Abnormal mitochondrial metabolism	6	1.81	*NDUFS7, ETFB, MYH7, NDUFAF5, ACADVL, NDUFA6*
0002157	Azotemia	5	1.76	*HADHB, ETFB, ACADVL, NDUFA6, PFKM*
0003394	Muscle spasm	5	1.7	*HADHB, MYH7, RYR1, ACADVL, PFKM*
0001635	Congestive heart failure	6	1.64	*TRPM4, HADHB, PPA2, ETFB, MYH7, RYR1*
0001254	Lethargy	6	1.63	*NDUFS7, HADHB, RYR1, NDUFAF5, ACADVL, NDUFA6*
0003236	Elevated creatine kinase concentration	7	1.59	*C1QBP, HADHB, ETFB, MYH7, RYR1, CAPN3, ACADVL*
0001943	Hypoglycemia	6	1.57	*NDUFS7, HADHB, ETFB, NDUFAF5, ACADVL, NDUFA6*
0001639	Hypertrophic cardiomyopathy	6	1.54	*NDUFS7, PPA2, MYH7, NDUFAF5, ACADVL, NDUFA6*
0001638	Cardiomyopathy	10	1.49	*C1QBP, NDUFS7, HADHB, PPA2, ETFB, MYH7, RYR2, NDUFAF5, ACADVL, NDUFA6*
0011675	Arrhythmia	8	1.48	*TRPM4, HADHB, PPA2, ETFB, MYH7, RYR1, RYR2, ACADVL*
0004372	Reduced consciousness confusion	7	1.48	*NDUFS7, HADHB, ETFB, RYR1, NDUFAF5, ACADVL, NDUFA6*
0004364	Abnormal nitrogen compound concentration	9	1.45	*C1QBP, HADHB, ETFB, MYH7, RYR1, CAPN3, ACADVL, NDUFA6, PFKM*
0001713	Abnormal cardiac ventricle morphology	8	1.31	*C1QBP, NDUFS7, HADHB, MYH7, RYR1, RYR2, NDUFAF5, ACADVL*

†- level of phenotype enrichment strength is obtained from STRING HPO analysis. Significance of enrichment was $p \leq 9.36E{-}06$.

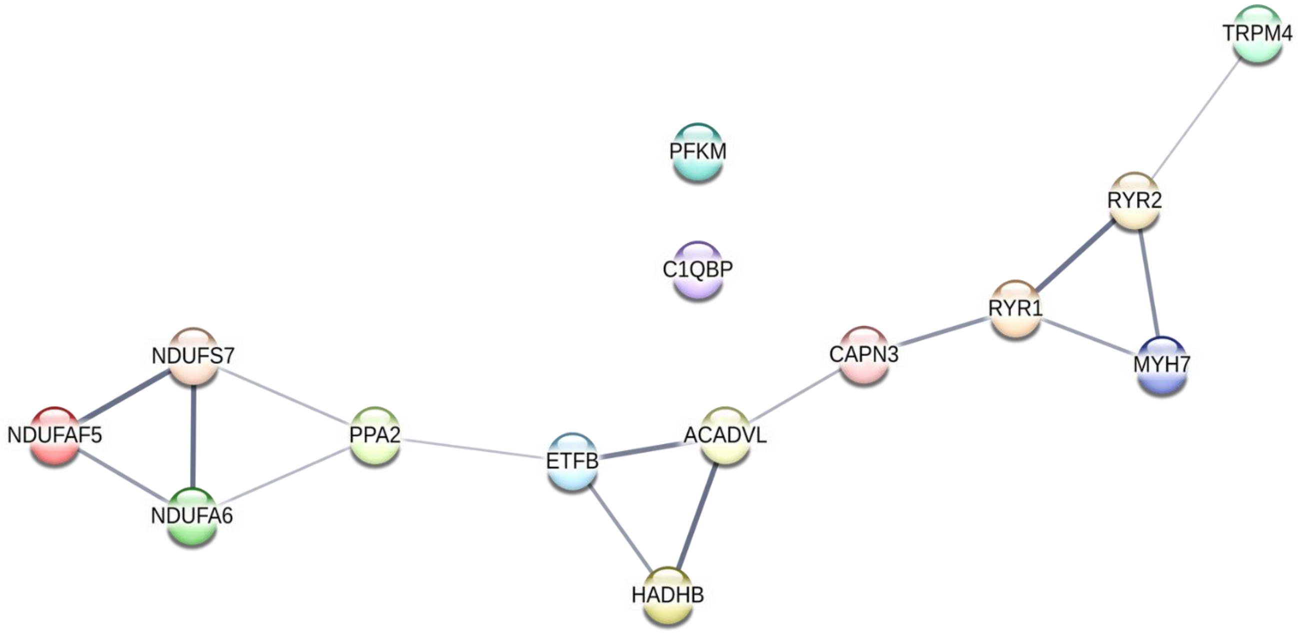

Figure S2. STRING protein-protein interaction results from proteins encoded by 14 genes carrying pathogenic variants in EHI cases. Of encoded proteins, 12 demonstrate functional and physical interactions. Protein-protein interaction enrichment indicated significant interactions among these proteins (p-value: 8.36E-10). Protein names are given in the center of the node; thickness of interconnecting lines indicates strength of interactions.

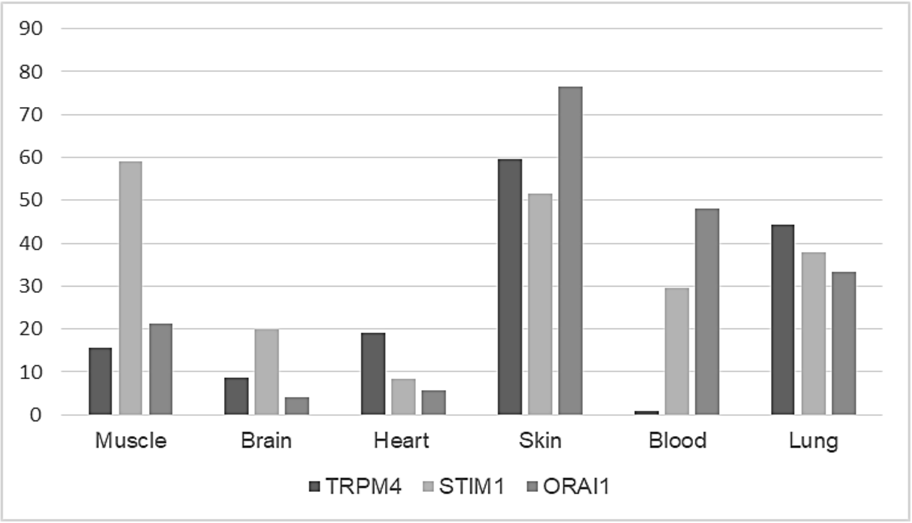

Figure S3. Tissue expression profile of *TRPM4* transcripts. *TRPM4* transcripts were compared to transcripts of genes, *STIM1* and *ORAI1*, encoding membrane proteins.

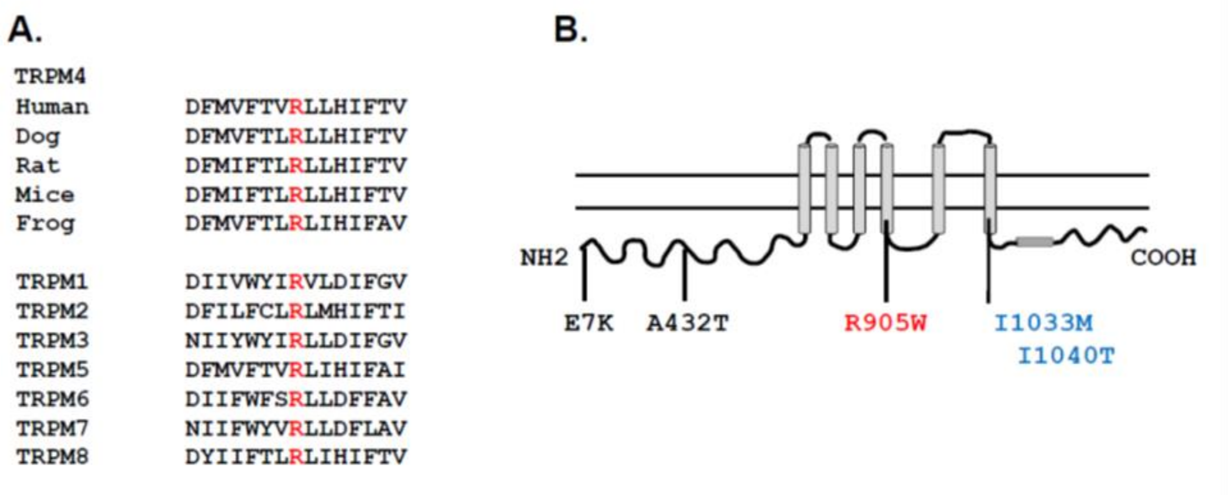

Figure S4. Amino acid alignment and locations of disease-associated variants within TRPM4. **A.** Arginine at 905 is highly conserved within TRPM4 of different species and between various subtypes of human TRPM channels. **B.** The p. Arg905Trp variant locates in the S4 transmembrane domain of TRPM4. The pathogenic variants p.Glu7Lys and p.Ala432Thr (in black) associated with progressive familial heart block [6,7] are located in the N-terminal domain. The pathogenic variants p.Ile1033Met and p.Ile1040Thr (in blue) associated with progressive symmetric erythrokeratodermia [8] are located in the S6 transmembrane domain of TRPM4.

Functional study results

TRPM4 currents from WT and R905W channels were recorded in excised patches in order to control exposure to activating Ca^{2+} and PIP_2 (Figure S5). Patches excised into a solution of elevated Ca^{2+} (1.8 mM) activated rapidly and responded to different levels of Ca^{2+}. Both WT and R905W channels were stably activated by PIP_2 (Figure S5.C) and exhibited stable Ca^{2+} sensitivity in the continued presence of PIP_2 (Figure S5. A, B).

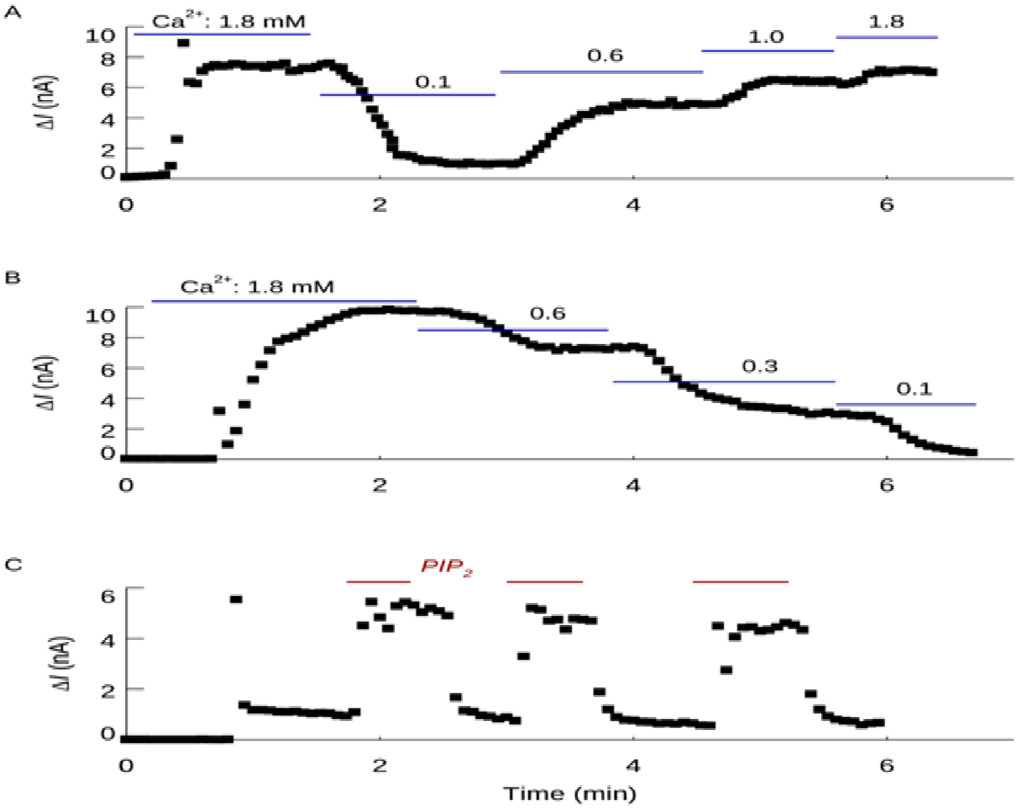

Figure S5. Electrophysiology of a wild-type (WT) and R905W TRPM4. ***A***. Time course of WT-TRPM4 currents in an excised patch during exposure to different Ca^{2+} concentrations (1.8, 0.1,0.6, 1.0 and 1.8 mM, indicated by blue bars) and 8 uM PIP_2 (continuous exposure). Each point represents a voltage-ramp of indicated magnitude (ΔI), as described in Methods. **B**. Sensitivity of excised R905W-TRPM4 channels to Ca^{2+} in the continued presence of 8 µM PIP_2. **C**. Currents from R905W-TRPM4 channel excised patch during repeated exposures to 8 uM PIP_2 in the continued presence of 1 mM Ca^{2+} demonstrate stable sensitivity to PIP_2 throughout the run.

References

1. Ren, M.; Sambuughin, N.; Mungunshukh, O.; Edgeworth, D.B.; Hupalo, D.; Zhang, X.; Wilkerson, M.D.; Dalgard, C.L.; O'Connor, F.G.; Deuster, P.A. Genome-wide analysis of exertional rhabdomyolysis in sickle cell trait positive african americans. *Genes* **2024**, *15*, article 408, doi:10.3390/genes15040408.
2. Sambuughin, N.; Swietnicki, W.; Techtmann, S.; Matrosova, V.; Wallace, T.; Goldfarb, L.; Maynard, E. KBTBD13 interacts with Cullin 3 to form a functional ubiquitin ligase. *Biochem Biophys Res Commun* **2012**, *421*, 743–749, doi:10.1016/j.bbrc.2012.04.074.
3. Mungunsukh, O.; Lee, Y.H.; Bottaro, D.P.; Day, R.M. The hepatocyte growth factor isoform NK2 activates motogenesis and survival but not proliferation due to lack of Akt activation. *Cell Signal* **2016**, *28*, 1114–1123, doi:10.1016/j.cellsig.2016.05.012.

4. Watkins, H.; Rosenzweig, A.; Hwang, D.-S.; Levi, T.; McKenna, W.; Seidman, C.E.; Seidman, J.G. Characteristics and prognostic implications of myosin missense mutations in familial hypertrophic cardiomyopathy. *NEJM* **1992**, *326*, 1108–1114, doi:10.1056/nejm199204233261703.

5. Zigova, M.; Bernasovska, J.; Boronova, I.; Mydlarova Blascakova, M.; Kmec, J. Finding the candidate sequence variants for diagnosis of hypertrophic cardiomyopathy in East Slovak patients. *J Clin Lab Anal* **2017**, *32*, article e22303, doi:10.1002/jcla.22303.

6. Kruse, M.; Schulze-Bahr, E.; Corfield, V.; Beckmann, A.; Stallmeyer, B.; Kurtbay, G.; Ohmert, I.; Schulze-Bahr, E.; Brink, P.; Pongs, O. Impaired endocytosis of the ion channel *TRPM4* is associated with human progressive familial heart block type I. *J Clin Invest* **2009**, *119*, 2737–2744, doi:10.1172/jci38292.

7. Liu, H.; El Zein, L.; Kruse, M.; Guinamard, R.; Beckmann, A.; Bozio, A.; Kurtbay, G.; Mégarbané, A.; Ohmert, I.; Blaysat, G.; et al. Gain-of-function mutations in *TRPM4* cause autosomal dominant isolated cardiac conduction disease. *Circ Cardiovasc Genet* **2010**, *3*, 374–385, doi:10.1161/circgenetics.109.930867.

8. Wang, H.; Xu, Z.; Lee, B.H.; Vu, S.; Hu, L.; Lee, M.; Bu, D.; Cao, X.; Hwang, S.; Yang, Y.; et al. Gain-of-function mutations in *TRPM4* activation gate cause progressive symmetric erythrokeratodermia. *J Invest Dermatol* **2019**, *139*, 1089–1097, doi:10.1016/j.jid.2018.10.044.
